# Plasmapheresis in neurological disorders: six years experience from University Clinical center Tuzla

**DOI:** 10.12688/f1000research.11841.1

**Published:** 2017-07-26

**Authors:** Osman Sinanović, Sanela Zukić, Adnan Burina, Nermina Pirić, Renata Hodžić, Mirza Atić, Mirna Alečković-Halilović, Enisa Mešić

**Affiliations:** 1 Department of Neurology, University Clinical Centre Tuzla, University of Tuzla, Tuzla, Bosnia and Herzegovina; 2 Division of Nephrology and Dialysis, Department of Internal Diseases, University Clinical Centre Tuzla, University of Tuzla, Tuzla, Bosnia and Herzegovina

**Keywords:** plasmapheresis, therapeutic plasma exchange, neurological disorders, myasthenia gravis, Guillain-Barré syndrome, demyelinating diseases, chronic inflammatory demyelinating polyneuropathy

## Abstract

Background: Therapeutic plasma exchange (TPE) is an extracorporeal blood purification technique that is designed to remove substances with a large molecular weight. The TPE procedure includes removal of antibodies, alloantibodies, immune complexes, monoclonal protein, toxins or cytokines, and involves the replenishment of a specific plasma factor. The aim of the study was to describe the clinical response to TPE in various neurological patients, and to assess the clinical response to this therapy.

Methods: The study was retrospective. We analyzed the medical records of 77 patients who were treated at the Department of Neurology, University Clinical Center (UCC) Tuzla from 2011 to 2016.

Results: 83 therapeutic plasma exchanges were performed in the 77 patients. There was a slight predominance of male patients (54.5%), with an average age of 51±15.9 years. The most common underlying neurological diseases were Guillain–Barré syndrome (GBS) (37.7%), then chronic inflammatory demyelinating polyneuropathy (CIDP) (23.4%), multiple sclerosis (MS) (11.7%) and myasthenia gravis (10.4%). Less frequent neurological diseases that were encountered were paraneoplastic polyneuropathies (5.2%), neuromyelitis optica (also known as Devic’s disease) (3.9%), motor neuron disease (3.9%), polymyositis (2.6%) and multifocal motor neuropathy (1.2%).

Conclusions: Six years experience of therapeutic plasma exchange in neurological patients in our department have shown that, following evidence-based guidelines for plasmapheresis, the procedure was most effective in patients with GBS, CIDP and myasthenia gravis.

## Background

Therapeutic plasma exchange (TPE) is an extracorporeal blood purification technique designed for the removal of large molecular weight substances. The basic premise of the treatment is that removal of these substances will allow for the reversal of the pathologic processes related to their presence ^1^. In Asia and Australia TPE is most commonly used for treatment of digestive system diseases, whereas in Europe and USA neurologic disorders prevail ^2^. While first experiences with TPE relate to acute life-threatening conditions, such as treatment of Guillain-Barre syndrome (GBS) or myasthenic crisis, therapeutic success hass also been shown for chronic diseases where immunosuppressive therapy is often required for long-term management ^3^. The TPE procedure includes removal of antibodies, alloantibodies, immune complexes, monoclonal protein, toxins or cytokines, and involves the replenishment of a specific plasma factor ^4– 7^.

The aim of the study was to describe the clinical response to TPE in various neurological patients, and to assess the clinical response to this therapy.

## Methods

This study is retrospective, and examines medical records of patients who were treated at the Department of Neurology, University Clinical Center (UCC) Tuzla from January 2011 to December 2016. We recorded the patient demographics, the neurological findings of patients on admission, the diagnosis that prompted treatment with TPE, comorbidities, and any medical complications that took place. Hematological parameters including blood counts, serum proteins, electrolytes and coagulation profiles were monitored after every TPE. The neurological state of patients and the recovery and outcome of therapy were assessed again when discharged. The study received institutional ethical approval from the University Clinical Center Tuzla, and also written informed consent was obtained from all patients who were treated with TPE.

## Results

83 therapeutic plasma exchanges were performed, in 77 patients, over the course of six years (2011 – 2016) at the Department of Neurology, University Clinical Center (UCC) Tuzla. Some of the patients received more than one course of plasmapheresis. There was a slight predominance of male patients (54.5%), with an average age of 51±15.9 years. The youngest patients were 16, and the oldest 78 ( Table 1). Most patients were from the Tuzla Canton, but 28 of them were from other cantons of the Bosnia and Herzegovina, and one patient was from Croatia.

**Table 1.** Demographic and clinical characteristics of the neurological patients that were treated with therapeutic plasma exchange at the Department of Neurology, University Clinical Center (UCC) Tuzla.

*Patient characteristics*	
Sex (female/male)	35/42
Mean age (years)	51±15.9
Average number of plasmapheresis sessions per patient	3 (every other day)
Neurological disease (n/%) Guillain–Barré syndrome Chronic inflammatory demyelinating polyneuropathy Multiple sclerosis Myasthenia gravis Paraneoplastic polyneuropathy Neuromyelitis optica (Devic`s disease) Motor neuron disease Polymyositis Multifocal motor neuropathy	29/37.7 18/23.4 9/11.7 8/10.4 4/5.2 3/3.9 3/3.9 2/2.6 1/1.2
Most common comorbidity Hypertension Diabetes Cancer Heart disease Thrombovascular event	27 9 3 3 3

TPE is usually carried out across three sessions. In 27 patients, it was carried out in five sessions, and in one case of severe poliradiculoneuritis in a young patient with tetraplegia, seven sessions were carried out. The most common underlying neurological disease poliradiculoneuritis, Guillain-Barré syndrome (GBS), presenting in 29 patients. These patients had a very good response to the therapy, and a good recovery of motor strength was observed. All patients with paraparesis or quadriparesis recovered some movement, even those with quadriplegia. All patients continue physical therapy in stationary conditions.

Two patients had complications, including deep vein thrombosis, but after treatment continued with physical therapy. One patient developed pneumonia, due to immobility (hypostatic pneumonia), not related to TPE. Unfortunately, one patient died after the third session of plasmapheresis.

18 patients with chronic inflammatory demyelinating polyneuropathy (CIDP) underwent TPE. Due to the disease being chronic, improvement generally was slower than in the acute form of demyelinating polyneuropathy. All patients felt recovery of motor strength and their sensory ability increased, after treatment. In some of these patients TPE had been repeated over the years.

Patients with severe forms of myasthenia gravis, with generalized muscle weakness, and in some of them respiratory failure, also underwent TPE. All these procedures had no complications, and all the patients recovered motor strength, except one, where no benefits were observed.

TPE was also carried out on patients with demyelinating diseases, including nine with chronic progressive form of MS and three with neuromyelitis optica. Patients who were treated with TPE had progressive forms of MS, with a high score on the Expanded Disability Status Scale (EDSS > 7.0), and we accomplished improvements in symptoms such as tremors, spasms or paresthesias, and there was slightly improvement in motor strength. One MS patient in the progressive stage of disease died. One patient with neuromyelitis optica died, but after the treatment, on palliative care. The other two patients with Devic’s disease, with spastic quadriplegia, managed to take a few steps after treatment with an orthopedic tool after discharge, during physical therapy.

Significant improvements after TPE were observed in two patients with polymyositis, including better mobility and pain reduction, and in four patients with paraneoplastic syndromes improvements in motor strength and reduced paresthesia were observed. One of the patients had a diagnosis of cerebellar paraneoplastic disorder, caused by breast cancer, with distal weakness, tremor, ataxia and loss of perception, and inability to walk. After three courses of plasmapheresis, of five sessions each, the patient was able to walk for short distances with help. A patient with multifocal motor neuropathy had severe muscle weakness and milder atrophy, but after treatment he noticed improvement in muscle strength. However, in three patients with motor neuron disease, plasmapheresis had no effect.

Alongside the TPE, patients were receiving treatment for their underlying neurological condition (steroids, immunosuppressive agents). Also, it is important to emphasize that all patients continued with physical therapy. A good outcome of the procedure was observed in 87% of patients (improvements were registered in 25 out of 29 GBS patients, in 18 patients with CIDP, 8 with MS, 7 with myasthenia gravis, 4 with paraneoplastic disorders, 2 with Devic’s disease, 2 with polymyositis and in one patient with multifocal motor neuropathy). Only one complication was observed (pneumotorax), but the patient fully recovered. Death was registered in three patients: two had severe, progressive forms of demyelinating disease and one patient with GBS experienced sudden death. The deaths were not directly related to plasmapheresis, but rather as results of complications associated with the disease.

Data of neurological patients that were treated with therapeutic plasma exchange, with demographic and clinical characteristics Click here for additional data file. Copyright: © 2017 Sinanović O et al. 2017 Data associated with the article are available under the terms of the Creative Commons Zero "No rights reserved" data waiver (CC0 1.0 Public domain dedication).

## Discussion

The American Academy of Neurology proposed an evidence-based guideline for plasmapheresis in neurological disorders. According to these recommendations, there is strong evidence that treatment is beneficial in severe forms of GBS (severe enough to impair independent walking or to require mechanical ventilation), and also as a short-term treatment for patients with CIDP ^8– 10^. Following these evidence-based guidelines, we treated 29 GBS patients with plasmapheresis (37.7%) and 18 CIDP patients (23.4%). Furthermore, according to these guidelines there was good evidence for treating polyneuropathy patients with plasmapheresis, and also that it had shown benefits as an adjunctive treatment in relapsing forms of MS ^11– 15^. We treated MS patients with progressive forms of the disease, and we only achieved a mild improvement of symptoms, but no significant improvement of EDSS scores.

The study by Láinez-Andrés *et al*. concluded that TPE proved to be an effective alternative treatment for diseases such as GBS, CIDP and myasthenia gravis. In comparative studies with intravenous immunoglobulin, the efficacy of both therapies is similar ^16^. A recent, large meta-analysis by Ortiz-Sales *et al* (2016) concluded that there is no evidence that either treatment is more effective or safe in the management of GBS and myasthenia gravis ^17^.

## Conclusion

Six years experience of therapeutic plasma exchange in neurological patients in our department have shown that, following evidence-based guidelines for plasmapheresis, the procedure was the most effective in patients with GBS, CIDP and myasthenia gravis. We did not record any significant complications associated with the procedure itself, only complications associated within the course of the patients’ neurological disease.

## Data availability

The data referenced by this article are under copyright with the following copyright statement: Copyright: © 2017 Sinanović O et al.

Data associated with the article are available under the terms of the Creative Commons Zero "No rights reserved" data waiver (CC0 1.0 Public domain dedication). 

**Dataset 1. Data of neurological patients that were treated with therapeutic plasma exchange, with demographic and clinical characteristics.**

doi, 10.5256/f1000research.11841.d169179 ^18^
