# Supplementary material for: Plasmapheresis in neurological disorders: six years experience from University Clinical center Tuzla
Source: F1000Res. 2017 Jul 26;6:1234. [Version 1] doi: 10.12688/f1000research.11841.1 (PMC5590081; doi:10.12688/f1000research.11841.1)
Supplement: Peer review history

# Peer review: Referee response for version 1

**DOI:** 10.5256/f1000research.12795.r25550

**Published:** 2017-09-06

**Authors:** Hidenori Matsuo

**Affiliations:** 1 Department of Clinical Research, Nagasaki Kawatana Medical Center, Nagasaki, Japan

The experience of therapeutic apheresis in Tuzia is described.

 The authors should discuss more the use of TPE in patients with polymyositis or motor neuron disease. According to guidelines, TPE in these diseases seems ineffective. Why did the authors perform TPE for the patients, and how do they think this effects polymyositis?

I have read this submission. I believe that I have an appropriate level of expertise to confirm that it is of an acceptable scientific standard.

# Peer review: Referee response for version 1

**DOI:** 10.5256/f1000research.12795.r24544

**Published:** 2017-08-29

**Authors:** David B. Vodusek

**Affiliations:** 1 Division of Neurology, University Medical Center, Ljubljana, Ljubljana, Slovenia

This report on a retrospective analysis of neurological patients treated with PE reflects the practice in a big neurological department in the years 2011-2016. As such it is intrinsically interesting as it provides data on neurological practice in an European region.

 It would add to the information if some additional general data were provided (population served by the department; number of patients treated per year)

 The outcome of PE treatment should be - if possible - described in more detail, and only results, no explanation should be given in the Results section ("..due to disease being chronic..."). Improvements as reported subjectively by patients, and those resulting in objective improvement in function, should be differentiated.

 In GBS, CIDP, MG and whenever else possible the authors should describe when in the time course of the disease the PE was given, what were the deficits before PE, when was the improvement noted and what was the final outcome in terms of function.

 It is stated that the patients treated with PE had also immunosuppressive drugs - but it remains unclear whether all patients (GBS??), and which drugs, and whether the drug regime was changed during PE.

 It would be interesting to note why it was decided to give PE in MND.

 It would be best to describe all untoward effects of PE in one paragraph.

 It would be interesting to note whether IVIG has also been used in the department in the same time period.

I have read this submission. I believe that I have an appropriate level of expertise to confirm that it is of an acceptable scientific standard.
